# A new species of *Allostoma* (Platyhelminthes, Prolecithophora) from the coast of Japan

**DOI:** 10.3897/BDJ.14.e176933

**Published:** 2026-01-16

**Authors:** Nao Omi

**Affiliations:** 1 Unaffiliated, Houston, United States of America Unaffiliated Houston United States of America

**Keywords:** Pseudostomidae, Prolecithophora, Turbellaria, Asia, Japan

## Abstract

**Background:**

The genus *Allostoma* von Beneden, 1861 (Pseudostomidae Graff, 1904) consists of generally small, often conspicuously colored species. These organisms are typically found on hard bottoms, in gravel, or among algae in marine and brackish-water environments. The Pseudostomid taxon encompasses approximately 55 known species globally, spanning regions across Europe, Asia, and North and South America. In Japan, however, only three species of this taxon have been reported to date: *Allostoma durum* (Fuhrmann, 1896), *Allostoma densissimabursa* (Omi, 2020) comb. nov., and *Cylindrostoma monotrochum* (Graff, 1882).

**New information:**

Samples of seaweed and sand were collected from the coast of Shimane Prefecture in western Japan. Turbellarians were subsequently isolated and examined both in their live state and in histological sections. This investigation led to the description of a new species, *Allostoma matsueensis* sp. nov., characterized by a pear-shaped granular vesicle, non-sclerotized retractile penis, a narrow rigidly sclerotized conical spermatic duct, a supraterminal female pore, and a thin membraned bursa. The species further exhibits a yellow-to-pale brown body. This discovery constitutes the first record of the genus *Allostoma* from the western marine coast of Japan.

## Introduction

The prolecithophoran taxon Karling, 1940, is a group of small flatworms (typically <1 cm in length) that are usually spindle-shaped and often conspicuously colored (Karling 1978, Karling 1993, Noren and Jondelius 2002). This taxon encompasses five recognized families: Multipeniatidae Karling, 1940, Plagiostomidae Graff, 1882, Protomonotresidae Reisinger, 1924, Pseudostomidae Graff, 1904, and Scleraulophoridae Marcus, 1950 (Jondelius et al. 2001, Norén and Jondelius 1999). Representatives of the pseudostomidae exhibit distinct morphological features, including a common oral–genital pore, a capsulated brain, two paired eyes, a pharynx plicatus, and a reproductive system characterized by an ovary not fully separated from the vitellarium, often accompanied by female accessory organs and a vaginal pore (Cannon 1986, Karling 1940, Karling 1962, Karling 1993, Westblad 1955). The pseudostomid taxon currently comprises eleven genera: *Allostoma* Beneden, 1861, *Cylindrostoma* Ørsted, 1845, *Einarhelmins* Karling, 1993, *Euxinia* Graff, 1911, *Gonostomula* Westblad, 1955, *Monoophorum* Bohmig, 1890, *Pregermarium* Stirewalt, Ferguson & Kepner, 1942, *Pseudostomum* Schmidt, 1848, *Reisingeria* Westblad, 1955, *Thallagus* Marcus, 1951, and *Ulianinia* Levinsen, 1879 (Tyler et al. 2025a, Tyler et al. 2025b).

*Allostoma* species possess several key morphological features, including a ciliated groove, a dorsal ovary with an associated bursa, a spermatic duct, a vagina externa, a common oral–genital pore positioned near the rear end, and a capsulated brain (Beneden 1861, Cannon 1986, Karling 1993, Westblad 1955). Distribution records for the genus *Allostoma* include Europe, North America, South America, China, and Japan (Ma et al. 2014, Omi 2020, Tsuyuki et al. 2024, Tyler et al. 2025b).

This study presents the formal description of a new species of the genus *Allostoma*, based on morphological reconstructions made from histological sections and observations of living animals from the coast of the Sea of Japan.

## Materials and methods

Sand and seaweed samples were collected in 2020 from the Sea of Japan, specifically off the coast of Shimane Prefecture (35°33'29.00"N, 133°18'31.92"E). Sampling occurred at depths of 0.5 to 3 m using a plankton net. The new species was found in water where salinity exceeded 27‰, as determined by an EB-158P digital salinity meter (Eishin, Japan). Specimens were carefully sorted and removed from the substrates under a stereomicroscope using a pipette. A cohort of more than nine (*n* = 9) specimens of the species was initially studied alive using light microscopy.

The specimens were first anesthetized with an isotonic magnesium chloride solution before being fixed with 10% formalin/Ethanol, then dehydrated through a graded ethanol series and xylene, and embedded in Tissue-Tek® paraffin Wax II 60 (Sakura Finetek, Tokyo, Japan). Serial sections (3–8 µm) were cut in sagittal, frontal, and transverse planes from the paraffin-embedded material using a Leica RM2055 microtome (Leica Microsystems, Germany). Specimens PL-6487 and PL-6489 were stained with the Azan trichrome method, whereas the holotype PL-6488 was stained with Masson’s trichrome (Romeis 1989). For the Masson-stained specimen, Mayer’s hematoxylin was used instead of iron hematoxylin (following a protocol from the University of Queensland Histology Facility; The University of Queensland Histology Facility 2014), which resulted in nuclei appearing reddish-purple due to the subsequent aniline-blue staining step. Stained sections were examined and photographed with an Olympus BX51 microscope (Olympus, Tokyo, Japan). The sectioned specimens (NSMT-Pl 6486-6490) have been deposited at the National Museum of Nature and Science (Tokyo, Japan).

Line drawings were produced by tracing morphological features from high-resolution micrographs of live and fixed specimens. Digital images were captured using an Olympus BX51 microscope equipped with a digital camera. Image processing was limited to global adjustments (brightness, contrast, and sharpness) using Adobe Photoshop (Adobe Inc., USA) and ImageJ v1.53 (National Institutes of Health, Bethesda, MD, USA), without any modification of anatomical structures.

## Taxon treatments

### Allostoma matsueensis

Omi sp. nov.

0273E61A-6551-5E65-A3E3-1F0A40B99449

740415AD-9CC0-4073-BA8D-3547FA6BCACF

#### Materials

- **Type status:** Holotype. **Occurrence:** catalogNumber: PL-6488; occurrenceRemarks: specimen represented by serial histological sections; recordNumber: Br-6; recordedBy: Nao Omi; individualCount: 1; sex: hermaphrodite; lifeStage: adult; occurrenceStatus: present; occurrenceID: E457F203-E4C1-5ED4-B0C2-D1A4C7D76D69; **Taxon:** scientificName: *Allostoma matsueensis*; parentNameUsage: Prolecithophora, Pseudostomidae; kingdom: Animalia; phylum: Platyhelminthes; order: Prolecithophora; family: Pseudostomidae; genus: Allostoma; taxonRank: species; **Location:** continent: Asia; waterBody: Sea of Japan; islandGroup: the Japanese archipelago; country: Japan; countryCode: JP; stateProvince: Shimane; county: Matsue; municipality: Mihonoseki; locality: the coast of Matsue city, Shimane Prefecture, Japan; verbatimCoordinates: 35°33'29.0"N 133°18'31.9"E; decimalLatitude: 35.558056; decimalLongitude: 133.308867; georeferenceProtocol: GPS; **Identification:** identifiedBy: Nao Omi; **Event:** samplingProtocol: plankton net; eventDate: 6/20/2020; year: 2020; month: June; day: 20; habitat: marine; **Record Level:** type: PhysicalObject; language: en; institutionID: https://ror.org/04r8tsy16; institutionCode: NSMT; collectionCode: PL; ownerInstitutionCode: TNS; basisOfRecord: PreservedSpecimen; **Material Entity:** preparations: histological formalin-fixed, paraffin-embedded serial histological sections
- **Type status:** Paratype. **Occurrence:** catalogNumber: PL-6486; occurrenceRemarks: specimen represented by serial histological sections; recordNumber: Br-2; recordedBy: Nao Omi; individualCount: 1; sex: hermaphrodite; lifeStage: adult; occurrenceStatus: present; occurrenceID: CBFD9733-74A1-5C97-9324-131D17A98C9B; **Taxon:** scientificName: *Allostoma matsueensis*; parentNameUsage: Prolecithophora, Pseudostomidae; kingdom: Animalia; phylum: Platyhelminthes; order: Prolecithophora; family: Pseudostomidae; genus: Allostoma; taxonRank: species; **Location:** continent: Asia; waterBody: Sea of Japan; islandGroup: the Japanese archipelago; country: Japan; countryCode: JP; stateProvince: Shimane; county: Matsue; municipality: Mihonoseki; locality: the coast of Matsue city, Shimane Prefecture, Japan; verbatimCoordinates: 35°33'29.0"N 133°18'31.9"E; decimalLatitude: 35.558056; decimalLongitude: 133.308867; georeferenceProtocol: GPS; **Identification:** identifiedBy: Nao Omi; **Event:** samplingProtocol: plankton net; eventDate: 6/20/2020; year: 2020; month: June; day: 20; habitat: marine; **Record Level:** type: PhysicalObject; language: en; institutionID: https://ror.org/04r8tsy16; institutionCode: NSMT; collectionCode: PL; ownerInstitutionCode: TNS; basisOfRecord: PreservedSpecimen; **Material Entity:** preparations: formalin-fixed, paraffin-embedded serial histological sections
- **Type status:** Paratype. **Occurrence:** catalogNumber: PL-6487; occurrenceRemarks: specimen represented by serial histological sections; recordNumber: Br-4; recordedBy: Nao Omi; individualCount: 1; sex: hermaphrodite; lifeStage: adult; occurrenceStatus: present; occurrenceID: FB84E696-8515-54BF-85E1-EF51E9C8F239; **Taxon:** scientificName: *Allostoma matsueensis*; parentNameUsage: Prolecithophora, Pseudostomidae; kingdom: Animalia; phylum: Platyhelminthes; order: Prolecithophora; family: Pseudostomidae; genus: Allostoma; taxonRank: species; **Location:** continent: Asia; waterBody: Sea of Japan; islandGroup: the Japanese archipelago; country: Japan; countryCode: JP; stateProvince: Shimane; county: Matsue; municipality: Mihonoseki; locality: the coast of Matsue city, Shimane Prefecture, Japan; verbatimCoordinates: 35°33'29.0"N 133°18'31.9"E; decimalLatitude: 35.558056; decimalLongitude: 133.308867; georeferenceProtocol: GPS; **Identification:** identifiedBy: Nao Omi; **Event:** samplingProtocol: plankton net; eventDate: 6/20/2020; year: 2020; month: June; day: 20; habitat: marine; **Record Level:** type: PhysicalObject; language: en; institutionID: https://ror.org/04r8tsy16; institutionCode: NSMT; collectionCode: PL; ownerInstitutionCode: TNS; basisOfRecord: PreservedSpecimen; **Material Entity:** preparations: formalin-fixed, paraffin-embedded serial histological sections
- **Type status:** Paratype. **Occurrence:** catalogNumber: PL-6489; occurrenceRemarks: specimen represented by serial histological sections; recordNumber: Br-8; recordedBy: Nao Omi; individualCount: 1; sex: hermaphrodite; lifeStage: adult; occurrenceStatus: present; occurrenceID: AA3FDB8C-2E6D-5371-9B22-F9A666EA3E0E; **Taxon:** scientificName: *Allostoma matsueensis*; parentNameUsage: Prolecithophora, Pseudostomidae; kingdom: Animalia; phylum: Platyhelminthes; order: Prolecithophora; family: Pseudostomidae; genus: Allostoma; taxonRank: species; **Location:** continent: Asia; waterBody: Sea of Japan; islandGroup: the Japanese archipelago; country: Japan; countryCode: JP; stateProvince: Shimane; county: Matsue; municipality: Mihonoseki; locality: the coast of Matsue city, Shimane Prefecture, Japan; verbatimCoordinates: 35°33'29.0"N 133°18'31.9"E; decimalLatitude: 35.558056; decimalLongitude: 133.308867; georeferenceProtocol: GPS; **Identification:** identifiedBy: Nao Omi; **Event:** samplingProtocol: plankton net; eventDate: 6/20/2020; year: 2020; month: June; day: 20; habitat: marine; **Record Level:** type: PhysicalObject; language: en; institutionID: https://ror.org/04r8tsy16; institutionCode: NSMT; collectionCode: PL; ownerInstitutionCode: TNS; basisOfRecord: PreservedSpecimen; **Material Entity:** preparations: formalin-fixed, paraffin-embedded serial histological sections
- **Type status:** Paratype. **Occurrence:** catalogNumber: PL-6490; occurrenceRemarks: specimen represented by serial histological sections; recordNumber: Br-12; recordedBy: Nao Omi; individualCount: 1; sex: hermaphrodite; lifeStage: adult; occurrenceStatus: present; occurrenceID: 9A84CA47-00CC-5580-AFC4-A354FC1A4C12; **Taxon:** scientificName: *Allostoma matsueensis*; parentNameUsage: Prolecithophora, Pseudostomidae; kingdom: Animalia; phylum: Platyhelminthes; order: Prolecithophora; family: Pseudostomidae; genus: Allostoma; taxonRank: species; **Location:** continent: Asia; waterBody: Sea of Japan; islandGroup: the Japanese archipelago; country: Japan; countryCode: JP; stateProvince: Shimane; county: Matsue; municipality: Mihonoseki; locality: the coast of Matsue city, Shimane Prefecture, Japan; verbatimCoordinates: 35°33'29.0"N 133°18'31.9"E; decimalLatitude: 35.558056; decimalLongitude: 133.308867; georeferenceProtocol: GPS; **Identification:** identifiedBy: Nao Omi; **Event:** samplingProtocol: plankton net; eventDate: 6/20/2020; year: 2020; month: June; day: 20; habitat: marine; **Record Level:** type: PhysicalObject; language: en; institutionID: https://ror.org/04r8tsy16; institutionCode: NSMT; collectionCode: PL; ownerInstitutionCode: TNS; basisOfRecord: PreservedSpecimen; **Material Entity:** preparations: formalin-fixed, paraffin-embedded serial histological sections

#### Description

**Figure 1.**
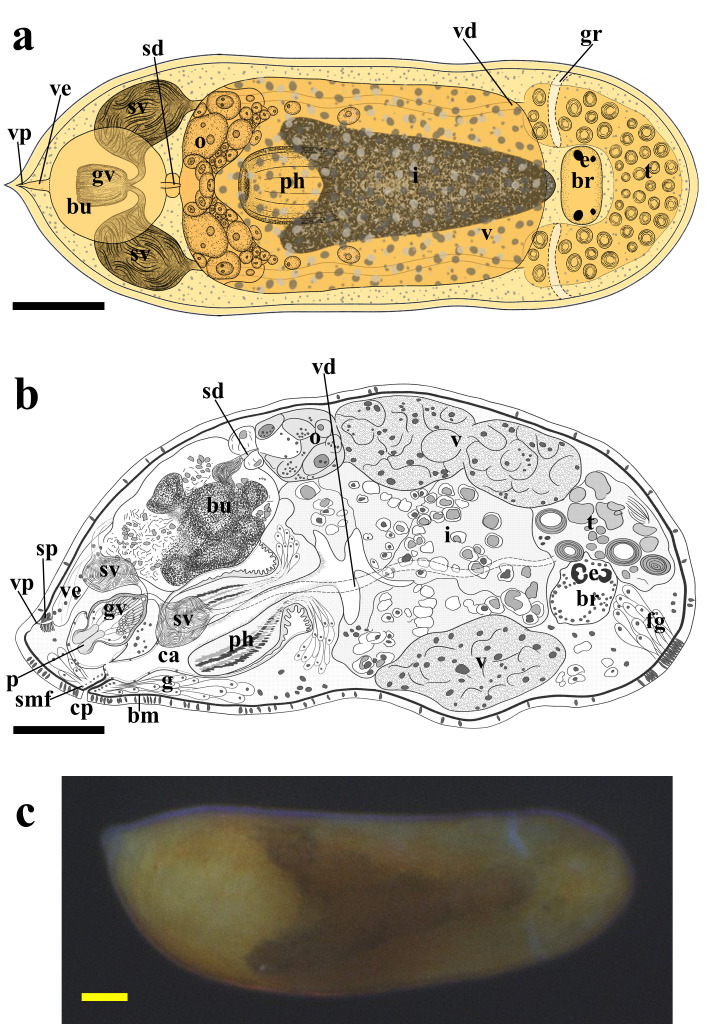
*Allostoma matsueensis* sp. nov. **a** frontal reconstruction of the holotype (PL-6488); **b** sagittal reconstruction of the same specimen; **c** photograph of the living holotype. Scale bar, 100 µm. Abbreviations: bm: basement mambrane; bu: bursa; br: brain; cp: common oral–genital pore; e: eye; fg: frontal gland; g: gland; gr: groove; i: intestine; gv: granular vesicle; o: ovary; p: penis; ph: pharynx; sd: spermatic duct; smf: sphincter muscle fiber; t: testis; v: vitellarium; ve: vagina externa; vp: vaginal pore; vd: vas deferens; sv: seminal vesicle.

**Figure 2.**
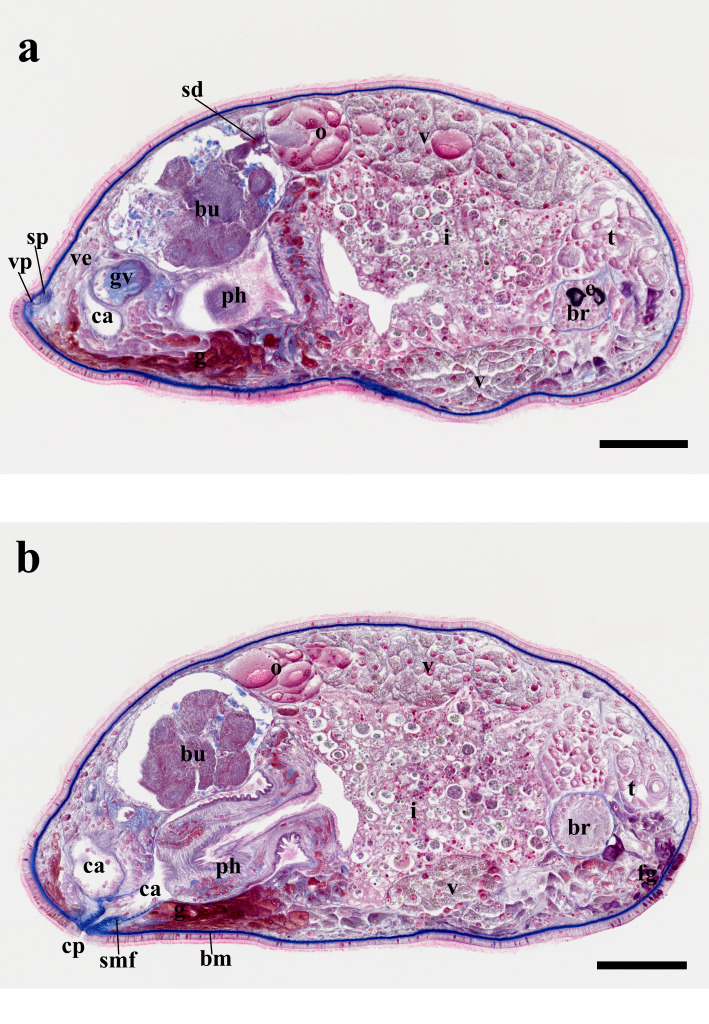
*Allostoma matsueensis* sp. nov. **a, b** general view of the holotype (PL-6488). Scale bars: 100 µm. Staining method: Masson’s trichrome. Abbreviations: bm: basement mambrane; br: brain; bu: bursa; ca: common atrium; cp: common oral–genital pore; e: eye; fg: frontal gland; g: gland; i: intestine; gv: granular vesicle; o: ovary; ph: pharynx; sd: spermatic duct; smf: sphincter muscle fiber; t: testis; v: vitellarium; ve: vagina externa; vp: vaginal pore.

**Figure 3.**
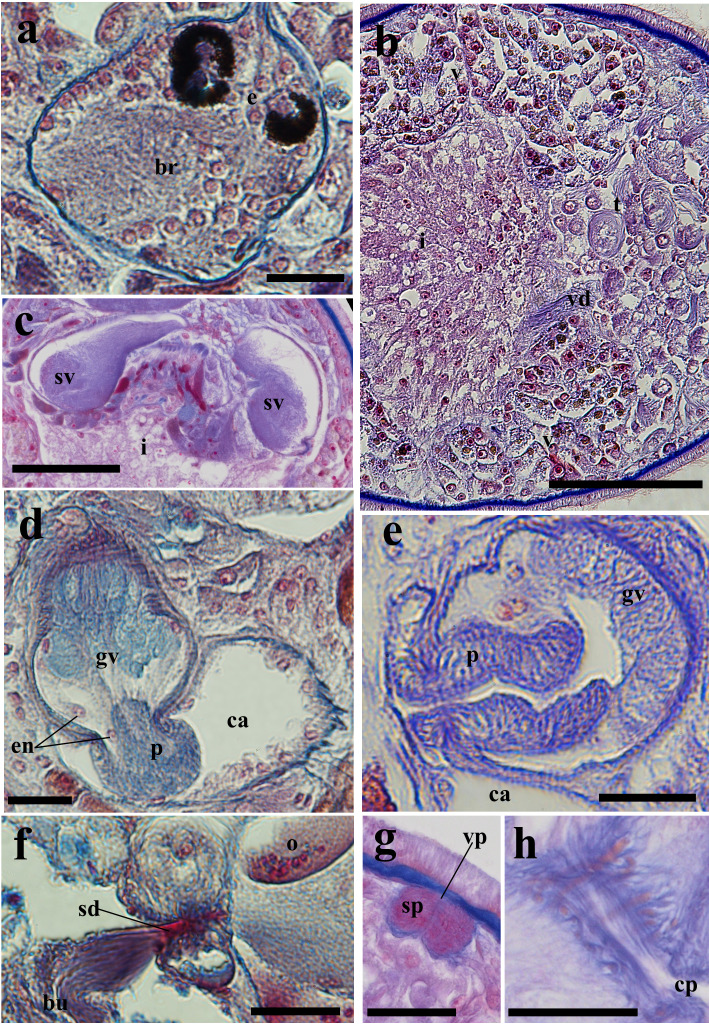
*Allostoma matsueensis* sp. nov. **a** details of the brain and eyes (PL-6488); **b** vas deferens (PL-6488); **c** paired seminal vesicle of the paratype (PL-6489); **d** male copulatory organ (PL-6488); **e** male copulatory organ of the paratype (PL-6487); **f** spermatic duct (PL-6488); **g** sphincter adjacent to the vaginal pore (PL-6489); **h** sphincter at the common oral–genital pore (PL-6487). Sections: b and d–f, sagittal sections; c, frontal section. Scale bars: 100 µm (b, c) and 25 µm (a, d, e, f, g). Staining methods: Masson’s trichrome (a, b, d, f) and Azan trichrome (c, e, g). Abbreviations: br: brain; bu: bursa; ca: common atrium; e: eye; en: epithelial nuclei; i: intestine; gv: granular vesicle; o: ovary; p: penis; sd: spermatic duct; sp: sphincter; t: testis; v: vitellarium; vd: vas deferens; sv: seminal vesicle.

*Allostoma matsueensis* exhibits a short, cylindrical body form, measuring 884 ± 132.9 µm in length and 316 ± 62.9 µm in width (*n* = 9), based on measurements taken from fully extended live specimens (Fig. 1c). The anterior end is rounded with a single ciliated groove near the brain, while the posterior end is slightly tapered. The species is characterized by a yellow to pale brown body with pigmentation present in the body parenchyma, and a brown intestine (Fig. 1a, c). The frontal gland is present on the ventral side of the anterior end and both cyanophil and erythrophil (Fig. 2b). Two pairs of black-pigmented eyes are located anteriorly, partially embedded in the brain capsule. The posterior eyes are heart-shaped (two lenses), and the anterior eyes are round (one lens) (Fig. 1a, b, Fig. 2a, Fig. 3a). The brain is encapsulated, positioned posterior to the eyes and testis, with the nuclei layer directly underlying the extracellular-matrix (ECM) membrane (Fig. 1b, Fig. 2a, b, Fig. 3a).

The pharynx is positioned posterior to the intestine, near the posterior body end. The pharynx narrows and becomes folded proximally, and corresponds to a pharynx variabilis, consistent with other prolecithophorans. Two types of pharyngeal glands are present: (1) an erythrophil granular gland type and (2) a cyanophil smooth-textured gland type. The nuclei of both gland types are clustered around the proximal pharyngeal base, whereas their glandular extensions and secretory regions continue distally toward the distal end of the pharynx. It comprises circular, longitudinal, and radial muscles organized into inner and outer muscular layers (muscularis interna and externa) and is ciliated on both internal and external surfaces (Fig. 1 , Fig. 2b). The esophagus is short (Fig. 2b).

The pharynx and penis open into a common atrium. The atrium is lined with ciliated epithelial cells, the pharyngeal and genital regions of the atrium are partially delimited by a zone of dense extracellular-matrix (ECM) fibers continuous with the basement membrane, without forming a complete partition (Fig. 1b, Fig. 2a, b, Fig. 3d, e). The thin ECM-membraned intestine lies posterior to the testis (Fig. 1, Fig. 2). The common oral–genital pore is surrounded by erythrophil granular glands and is circular muscle fibers forming a sphincter; these fibers are distributed along the length of the common pore and interdigitate with the basement membrane, rather than forming a single sharply delimited muscular ring in one plane of the section. In individual sections these fibers appear as discrete profiles embedded within the ECM (Fig. 1b, Fig. 2b, Fig. 3h).

Regarding the male system, the testis is located near the anterior end (Fig. 1b, Fig. 2a). Two vasa deferentia extend from the testis, passing laterally through the intestine to connect with the paired seminal vesicles (Fig. 1a, Fig. 3b). The seminal vesicles subsequently join the granular vesicle via a narrow tube (Fig. 1a, b, Fig. 3c).

The male copulatory organ is located posterior to the pharynx and consists of a pear-shaped granular vesicle and a thick, non-sclerotized penis capable of retracting into or extending beyond the granular vesicle (i.e., flexible) (Fig. 1b, Fig. 2a, Fig. 3d, e). In the only specimen with a fully extended papilla (PL-6488), the penile papilla measured 42.3 µm in length, and the granular vesicle measured 92.3 µm in length, with maximum widths of 45.2 µm (proximal lobe) and 67.3 µm (distal lobe) (Fig. 3 d). The granular vesicle is encased in circular and longitudinal muscle fibers (Fig. 3d). Its proximal region contains cyanophil elongate and granular bodies and connecting fibers leading to the penis occupying the distal region (Fig. 3d). The penis is observed either retracted within the granular vesicle or externally protruded (Fig. 1b, Fig. 3d, e).

The female reproductive system contains a long ovary delimited by a thin membrane, which crosses the midline posterior to the intestine and connects to the vitellarium at both ends (Fig. 1a,b, Fig. 2a, b). The vitellarium is an unpaired structure, extending dorsally from the posterior boundary of the testis to the ovary, and partially envelops the ovary (Fig. 1a, b, Fig. 2a).

A large bursa, delimited by a thin membrane, occupies the posterodorsal region and contains multiple sperm masses (Fig. 1a, b, Fig. 2a, b). The vagina externa opens into the bursa and extends to the supraterminal vaginal pore; a sphincter is present near the pore (Fig. 1b, Fig. 2a, Fig. 3g). From the proximal part of the bursa, the sclerotized and conical spermatic duct, measuring 21.4 µm in length and tapering from 8 µm proximally to 2.5 µm distally, traverses a thin-membranous zone between the ovary and bursa and connects to an ovarian syncytial region, a multinucleated cytoplasmic mass located within the ovarian basement membrane and adjacent to the developing oocyte (Fig. 1b, Fig. 2a, Fig. 3f). No epithelial continuity or duct-like lumen between this region and the spermatic duct could be observed (Fig. 3f).

#### Diagnosis

*Allostoma matsueensis* sp. nov. is diagnosed based on the holotype (NSMT-Pl 6488) and paratypes (NSMT-Pl 6486–6490) by the following combination of characters: a short cylindrical body, measuring 884 ± 132.9 µm in length and 316 ± 62.9 µm in width (n = 9); body yellow to pale brown, with pigments occurring in the body parenchyma; granular vesicle pear-shaped (holotype: length 92.3 µm; proximal width 45.2 µm; distal width 67.3 µm); penis thick, non-sclerotized, and retractile, with a penile papilla 42.3 µm long in the holotype; bursa delimited by a thin membrane; spermatic duct narrow and sclerotized conical mouthpiece (holotype: length ca. 21.4 µm; width ca. 2.5–8 µm); ovary unpaired and crossing the midline; vaginal pore supraterminal, and vagina externa surrounded by a thin ECM membrane and a sphincter positioned adjacent to the vaginal pore. The new species is most similar to *Allostoma durum*, but differs in having a non-conical penis, a rigidly sclerotized (vs soft-walled) spermatic duct, a supraterminal (vs dorsal) female pore, and the absence of penile glands (Karling 1940, Omi 2020, Westblad 1955).

#### Etymology

The name is an adjective referring to the geographic origin, Matsue City, located within Shimane Prefecture, Japan.

#### Distribution

The coasts of Matsue City, Shimane Prefecture, Japan.

#### Notes

##### Habitat

Intertidal and shallow subtidal zones (0.5–3 m depth), found among sand and seaweed substrates.

##### Taxonomic remarks

The genus *Enterostomula* Reisinger, 1926 has been regarded as a junior synonym of *Allostoma* von Beneden, 1861 since the comprehensive revision by Westblad 1955, who concluded that the diagnostic characters previously used to separate the two genera do not justify generic distinction. Westblad explicitly stated that *Allostoma* and *Enterostomula* “must be made to compose one genus, viz. *Allostoma*” (Westblad 1955: 513). This taxonomic interpretation has subsequently been followed in later systematic treatments, in which former species of *Enterostomula* were transferred to *Allostoma* (e.g., Karling 1962, Karling 1993). Following this established taxonomic framework, the species originally described as *Enterostomula densissimabursa* Omi, 2020 (Omi 2020) is herein formally transferred to the genus *Allostoma* and is treated as *Allostoma densissimabursa* (Omi, 2020) comb. nov. This action constitutes the first explicit and formal establishment of this new combination in accordance with the ICZN. Accordingly, all references to *E. densissimabursa* in the present study are made under the combination *Allostoma densissimabursa* (Omi, 2020).

## Discussion

The majority of pseudostomids have been reported from European, Mediterranean, North and South American, and Hawaiian coasts. Asian records for the known pseudostomid taxa include *A. graffi* (de Beauchamp, 1913) from China, and three species from Japan: *A. durum* (Fuhrmann, 1896) in eastern Japan (Misaki), *A. densissimabursa* (Omi, 2020) in western Japan (Shinji Lake, a brackish environment), and *Cylindrostomum monotrochum* (Graff, 1882) in northern Japan (Rishiri Island) (Ma et al. 2014, Omi 2018, Omi 2020, Tsuyuki et al. 2024, Westblad 1955). The present study is therefore significant as it provides the first record of an *Allostoma* species from the western marine coast of Japan, whereas A. densissimabursa was described from a brackish inland lake in western Japan.

The new species, *A. matsueensis* sp. nov., is morphologically defined by a common oral–genital pore, a short droplet–shaped body, a pharynx variabilis, an encapsulated brain, an intestine with a thin ECM membrane, and an ovary that is incompletely separated from the vitellarium. Major diagnostic features also include a pear-shaped granular vesicle containing elongate and granular bodies, a thick non-sclerotized penis, a narrow conical sclerotized spermatic duct, a large bursa surrounded by a thin ECM membrane, a supraterminal vagina externa with a sphincter, and a midline-crossing unpaired ovary.

Species of *Allostoma* are defined by the presence of a ciliated groove, a dorsal ovary with an associated bursa, a spermatic duct, a vagina externa, a common oral–genital pore at the posterior end, together with an encapsulated brain. Within the pseudostomid taxon, *Allostoma* and the genus *Monoophorum* Bohmig 1890, share the common characteristics of a posterior oral–genital pore and a bursa. Critical distinction is provided by the female accessory organs: *Allostoma* possesses a vagina externa, while *Monoophorum* possesses a vagina atrialis (Beauchamp 1913, Böhmig 1890, Cannon 1986, Karling 1993, Westblad 1955).

The configuration of the two pairs of eyes, the encapsulated brain, and the presence of a frontal gland in Allostoma matsueensis sp. nov. conform to the general pattern reported for the genus *Allostoma* and related pseudostomids and are not considered diagnostic for the species (Cannon 1986, Karling 1993, Westblad 1955). Accordingly, species delimitation in *A. matsueensis* sp. nov. relies primarily on characters of the reproductive system.

Comparative remarks. A brief comparison with all described species of *Allostoma* highlights the distinctiveness of *A. matsueensis* sp. nov. Species such as *A. amoenum*, *A. catinosum*, and *A. pallidum* possess cylindrical or fusiform granular vesicles, whereas the new species possesses a pear-shaped vesicle (Beklemischev 1927, Beneden 1861, Karling 1962). *Allostoma crassicystiferum*, *A. hopkinsi*, and *A. uterinum* lack a sclerotized spermatic duct, whereas the new species possesses a narrow conical sclerotized spermatic duct (Karling 1993, Westblad 1952). Species including *A. neostiliferum*, *A. evelinae*, and *A. ronis* possess penile glands, whereas no penile glands are present in *A. matsueensis* sp. nov. (Karling 1993, Marcus 1948, Marcus 1954). In *A. oerstedi*, the paired ovaries lie laterally, whereas in the new species the ovary is unpaired and crosses the midline (Levinsen 1879). *Allostoma graffi* has a rounded flask-shaped vagina externa and lacks a body groove, whereas the new species possesses a ciliated groove and a non-flask-shaped vagina externa (Beauchamp 1913, Jones 1941, Ma et al. 2014).

*Allostoma matsueensis* sp. nov. is most closely allied with *A. durum*, as both possess two pairs of eyes, a vagina externa with a sphincter, a bursa with a spermatic duct (featuring a narrow mouthpiece connecting to the ovary), and a muscular, bulbous granular vesicle. The particularly close affinity between *A. matsueensis* sp. nov. and *A. durum* is further supported by the presence of a large bursa in both species, a feature illustrated for Japanese specimens of *A. durum* by Westblad (Westblad 1955). The female pore is supraterminal in *A. matsueensis* sp. nov., whereas in *A. durum* it is dorsal and positioned at approximately 82% of body length. The penis of *A. durum* is cone–shaped, unlike the penis of *A. matsueensis* sp. nov. The spermatic duct of *A. durum* is described by Karling as having a soft cuticular wall (Karling 1940), whereas the spermatic duct of the new species possesses a rigidly sclerotized conical mouthpiece. Additionally, *A. durum* bears penile glands and a ring–like strip and epithelial lining around the spermatic duct absent in the new species (Führmann 1898, Karling 1940, Westblad 1955). Comparison with *A. densissimabursa*, from a nearby brackish lake, also reveals major differences. *Allostoma densissimabursa* exhibits two black pigment bands and a thick bursa wall rich in extracellular matrix and muscle fibers (Omi 2020). In contrast, *A. matsueensis* sp. nov. lacks pigment bands and possesses a bursa with a thin ECM membrane wall. The coloration of *A. matsueensis* sp. nov. is uniformly yellow–pale brown, without the patterned pigmentation found in *A. densissimabursa* (Omi 2020).

## Supplementary Material

XML Treatment for Allostoma matsueensis
